# A randomized, placebo-controlled double-blinded comparative clinical study of five over-the-counter non-pharmacological topical analgesics for myofascial pain: single session findings

**DOI:** 10.1186/2045-709X-20-7

**Published:** 2012-03-21

**Authors:** Daniel Avrahami, Amanda Hammond, Ceara Higgins, Howard Vernon

**Affiliations:** 1 Toronto, ON, Canada; 2 Canadian Memorial Chiropractic College, 6100 Leslie Street, Toronto, ON M2H 3J1, Canada; 3 204 Parkmount, Toronto, ON M4J 4V6, Canada; 4 Lakeview Chiropractic Health and Wellness, 2B 3602 Taylor St. E, Saskatoon, SK S7H 5H9, Canada; 5 777 Woodbine Ave, Toronto, ON M4E 2J5, Canada

## Abstract

**Objectives:**

To investigate the effects of topical agents for the treatment of Myofascial Pain Syndrome (MPS) and Myofascial Trigger Point (MTRP).

**Methods:**

Subjects with an identifiable trigger point in the trapezius muscle, age 18-80 were recruited for a single-session randomized, placebo-blinded clinical study. Baseline measurements of trapezius muscle pressure pain threshold (PPT: by pressure algometer) along with right and left cervical lateral flexion (rangiometer) were obtained by a blinded examiner. An assessor blinded to the outcomes assessments applied one of 6 topical formulations which had been placed in identical plastic containers. Five of these topicals were proposed active formulations; the control group was given a non-active formulation (PLA). Five minutes after the application of the formula the outcome measures were re-tested. Data were analyzed with a 5-way ANOVA and Holms-adjusted t-tests with an alpha level of 0.05.

**Results:**

120 subjects were entered into the study (63 females; ages 16-82); 20 subjects randomly allocated into each group. The pre- and post-treatment results for pressure threshold did show significant intra-group increases for the Ben-Gay Ultra Strength Muscle Pain Ointment (BG), the Professional Therapy MuscleCare Roll-on (PTMC roll-on) and Motion Medicine Cream (MM) with an increased threshold of 0.5 kg/cm^2 ^(+/-0.15), 0.72 kg/cm^**2 **^(+/-0.17) and 0.47 Kg/cm^**2 **^(+/-0.19) respectively. With respect to the inter-group comparisons, PTMC roll-on showed significant increases in pressure threshold compared with Placebo (PLA) (p = 0.002) and Icy Hot Extra Strength Cream (IH) (p = 0.006). In addition, BG demonstrated significant increases in pressure threshold compared with PLA (p = 0.0003).

**Conclusions:**

With regards to pressure threshold, PTMC roll-on, BG and MM showed significant increases in pain threshold tolerance after a short-term application on a trigger points located in the trapezius muscle. PTMC roll-on and BG were both shown to be superior vs placebo while PTMC was also shown to be superior to IH in patients with trigger points located in the trapezius muscle on a single application.

CMCC Research Ethics Board Approval # 1012X01, 2011

## Introduction

Myofascial Pain Syndrome (MPS) is exceedingly common in Western society [1-3]. Vernon and Schneider note that MPS is thought to be the leading diagnosis among pain management specialists [4,5] and the leading diagnosis in pain patients reporting to general practitioners [4,6]. MPS can be viewed as a regional pain syndrome such as, neck, low back and upper quadrant pain syndromes. MPS can also be found focally in discrete painful sites known as Myofascial Trigger Point (MTrP). Locations of MTrP's have been characterized on clinical grounds throughout the musculoskeletal system, starting with the seminal work of Travell and Simons [6,7]. MTrP's have been described as active (clinically active with pain referral upon palpation) or latent (not clinically active, but tender on manual palpation) [7-9]. The presence of associated features such as "local twitch sign" and palpable taut bands is thought to confirm the presence of an active MTrP, while latent TP's may not present with the classic features.

Numerous treatments are currently available for MPS and MTrP's. Several systematic reviews have recently been published on a variety of treatments [4,10-14], including pharmacologic agents, physical agents, complementary and alternative therapies. One commonly used therapy is the application of topical agents to relieve pain [15-17]. These agents can be classified as pharmacologic or non-pharmacologic. The former category includes non-steroidal anti-inflammatory agents (such as ketaprofen), opioid agents, classical analgesic agents (such as lidocaine), novel analgesic agents such as capsaicin and rubefascient agents (containing salicylates or nicotinamides) [15-17]. Non-pharmacologic agents generally fall into the complementary and alternative medicine (CAM) category for MPS and MTrP treatments. They may contain putative analgesic agents or counterirritant agents and they may exert cold, hot or neutral effects.

Several studies have investigated the effect of such topicals in the treatment of osteoarthritis, particularly of the knee [18,19]. However, there appears to be a lack of studies investigating the effects of topical agents for the treatment of MPS or MTrP. A randomized, placebo-blinded clinical trial of non-pharmacological topical analgesics was conducted comparing leading national and professional brands in the treatment of a myofascial trigger point.

## Materials and methods

### Subjects

Subjects were selected from consecutive clinical presentations of patients for treatment in a multidisciplinary health clinic. Subjects aged 18-80, male or female, with or without neck pain were included. Subjects were excluded for the following reasons: acute pain presentation preventing comfortable participation and absence of a palpable tender spot in the right upper trapezius region. Subjects were informed of the nature of the experiment and consented to participate. This protocol was approved by the Research Ethics Board of the Canadian Memorial Chiropractic College.

### Randomization

A random number sequence was generated by an independent party and kept in the care of the clinic administrator until required for subject allocation. The randomization schedule was concealed from all other study staff prior to selection and prior to group allocation.

### Outcome measures

Pre- and post-intervention measures consisted of 1) tenderness at an MTrP in the upper trapezius and, 2) right and left cervical spine lateral flexion. Tenderness was measured using a manual pressure algometer and was reported in kg/cm^2 ^[20]. Once identified by palpation and marked on the skin, the MTrP was subjected to vertical pressure with the pressure algometer at the rate of 1 kg/cm^2 ^per second. The subject was instructed to indicate verbally when the first instance of tenderness was felt. A single trial was used. Right and left cervical spine lateral flexions were measured using a cervical rangiometer [21] and were reported in degrees. A single trial was used. Both measures have proven reliable and valid in the assessment of MTrP's [9,21-27]. Following the intervention, subjects were asked to rate their level of satisfaction on a verbal satisfaction scale (P = poor, F = fair, G = good, E = excellent).

### Interventions

Six topical products were tested: three were ointments, two were roll-on gels and one a non-medicinal placebo cream (PLA) which served as the control. The comparative topicals were Professional Therapy MuscleCare Roll-on^a ^(PTMC roll-on), Motion Medicine cream^b ^(MM cream), Bengay Ultra Strength Muscle Pain ointment^c ^(BG), Icy Hot Extra Strength Cream^d ^(IH), and Biofreeze roll-on gel^e ^(BF). All of these products were placed in identical 0.5 ounce white plastic screw top containers or 3 ounce generic white roll-on bottles. Only a coded letter was applied as a label. The master code for these products was kept with the clinic administrator and was unknown to all study participants and assessors.

### Procedures

**Figure 1:**
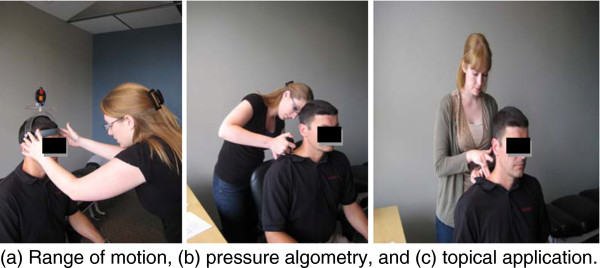
**(a) Range of motion, (b) pressure algometry, and (c) topical application**.

Subjects were seated erect in a comfortable ergonomic chair in a private room. Subjects wore a nose clip for the entire duration of the study so they could not smell any of the scents from the topical analgesics. Subjects were not allowed to view the application of the topical analgesics and they were not permitted to look at their shoulder during the entire process. Assessor #1 entered the room and palpated the subject's right shoulder in order to determine the presence and location of an MTrP in the upper trapezius muscle adjacent to the 7^th ^cervical vertebrae and the 1^st ^thoracic vertebrae. This was marked with a black dot. Assessor #1 exited the room and assessor #2 entered and performed the baseline testing of the outcome measures (Figure 1). The rangiometer was placed on the subject's head. From a neutral position, right and left active end-range lateral flexion measurements were recorded. The pressure algometer was applied over the marked trapezius trigger point for the baseline pressure reading. The subject was instructed to indicate when the pressure point was painful.

Following the initial outcome measurements, assessor #1 re-entered the room and applied one of the six samples according to the randomization schedule which was revealed only at that time in the area of the marked pressure point (Figure 1). The subject was instructed to stay seated in the chair with little head movement for five to seven minutes, following which, assessor #2 reassessed and recorded the pain and range of motion outcome measures. The study was conducted over a 7-day period.

### Analyses

Data were first analyzed descriptively--baseline, post-intervention and change scores. Change scores for each of the three measurements (Pressure pain threshold, Right and Left lateral flexion) were analyzed using a 5-way ANOVA. Statistically significant ANOVA's (p > 0.05) were then subjected to multiple t-tests with Holm's adjustment of the p-value. As such, the alpha levels were set at 0.007.

## Results

120 subjects were entered into the study, 20 in each group (63 females; ages 18-80). Fifty-six percent (56%) of patients presented with clinical shoulder or neck pain. Group demographics and clinical baseline values can be found in Table 1. There were no significant differences regarding group demographics and clinical baseline values.

**Table 1:** Group demographics and clinical baseline values

Variable	Group A	Group B	Group C	Group D	Group E	Group F	Statistic
AGE	48.5 (17.7)	48.5 (14.7)	47.9 (15.1)	47.1 (14.7)	45.0 (12.5)	51.1 (15)	NS F = 0.3 p = 0.89

GENDER	M = 8 F = 12	M = 8 F = 12	M = 11 F = 9	M = 11 F = 9	M = 11 F = 9	M = 7 F = 13	NS CSq = 4.0, p = 0.97

NECK and SHOULDER PAIN	Y = 11 N 9	Y = 12 N = 8	Y = 11 N = 9	Y = 10 N = 10	Y = 12 N = 8	Y = 11 N = 9	NS CSq = 2.2, p = 0.99

Pressure Pain Threshold (kg/in^2^)	4.5 (2.2)	4.6 (1.5)	4.9 (2.4)	4.2 (1.3)	4.1 (0.9)	4.5 (2.4)	NS F = 0.41 p = 0.83

The pre- and post-treatment results for pressure threshold are displayed in Table 2. ANOVA for intergroup differences was significant (F = 5.2, p = 0.002). Significant intra-group increases for BG, PTMC roll-on and MM cream were obtained with an increased threshold of 0.5 kg/cm^**2 **^(+/-0.15), 0.72 kg/cm^**2 **^(+/-0.17) and 0.47 Kg/cm^**2 **^(+/-0.19) respectively.

**Table 2:** Pressure pain thresholds.

Group	Pre-Tx (kg/cm^2^)	Post-Tx (kg/cm^2^)	Change (kg/cm^2^)	Intra-group Significance	Inter-group Significance*
**A**	4.5 (+/-0.48)	5.0 (+/-0.56)	0.5 (+/-0.15)	p = 0.002	A > C (p = 0.0003)

**B**	4.6 (+/-0.33)	4.5 (+/-0.35)	-0.45 (.12)	p = 0.37 NS	

**C**	4.9 (+/-0.53)	4.6 (+/-0.54)	-0.3 (+/-0.19)	p = 0.067 NS	

**D**	4.2 (+/-0.28)	4.96 (+/-0.39)	0.72 (+/-0.17)	p = 0.0002	D > C (p = 0.002) D > E (p = 0.006)

**E**	4.1 (+/-0.20)	4.4 (+/-0.32)	0.35 (+/-0.23)	p = 0.07 NS	

**F**	4.4 (+/-0.55)	4.9 (+/-0.59)	0.47 (+/-0.19)	p = 0.01	

A = Bengay Ultra Strength Muscle Pain ointment, B = Biofreeze roll-on gel, C = Placebo, D = Professional Therapy Muscle Care roll-on, E = Icy Hot Extra Strength Cream, F = Motion Medicine

*Bonferroni corrected p value = 0.007

With respect to the inter-group comparisons, PTMC roll-on showed significant increases in pressure threshold compared with PLA (p = 0.002) and IH (p = 0.006). In addition, BG demonstrated significant increases in pressure threshold compared with PLA (p = 0.0003).

The pre- and post-treatment results for right and left lateral flexion did not show significant increases in range of motion for any of the topical analgesics (Table 3).

**Table 3:** Right Lateral Flexion.

Group	Pre-Tx (Degrees)	Post-Tx (Degrees)	Change (Degrees)	Significance
**A**	39.4 (+/-3.3)	44.9 (+/-2.6)	5.45 (+/-1.7)	NS

**B**	45.9 (+/-2.3)	46.7 (+/-2.9)	.8 (+/-1.5)	NS

**C**	40.6 (+/-2.8)	40.1 (+/-2.4)	-0.45 (+/-1.1)	NS

**D**	44.4 (+/-2.9)	48.3 (+/-3.0)	3.95 (+/-1.6)	NS

**E**	46.9 (+/-2.7)	48.4 (+/-2.7)	1.4 (+/-1.5)	NS

**F**	44.5 (+/-1.7)	44.7 (+/-1.6)	0.2 (+/-1.1)	NS

A = Bengay Ultra Strength Muscle Pain ointment, B = Biofreeze roll-on gel, C = Placebo, D = Professional Therapy Muscle Care roll-on, E = Icy Hot Extra Strength Cream, F = Motion Medicine

Patient satisfaction ratings were high in each group and there were no significant differences between groups. There were no adverse reactions or complaints of pain aggravation reported in this study.

## Discussion

This study investigated the immediate effect of six different topical analgesic creams on pressure pain threshold at a single trapezius MTrP and on bilateral cervical lateral flexions. We found that the topical analgesics BG, PTMC roll-on and MM cream demonstrated significant and clinically important increases in pressure threshold when comparing short-term MTrP tenderness. In addition, PTMC roll-on was better than placebo and Icy Hot Extra Strength Cream in reducing short-term MTrP tenderness. BG was better than placebo at reducing short-term MTrP tenderness.

None of the groups demonstrated a statistically significantly increase in right or left lateral flexion.

The effectiveness of the topical analgesics that showed clinically significant improvements in cervical spine pressure threshold may be due to several factors. Eucalyptus oil, that was found in the PTMC solution, has been shown to transport active ingredients deep into the subcutaneous tissues [28,29]. Camphor and menthol, found in both BG and PTMC, have been proven to provide immediate pain relief [19,30,31]. Glucosamine sulfate, chondroitin sulfate, dimethyl sulfoxide and Boswellia serrata extract, found in the PTMC roll-on formulation, have been shown to improve circulation and reduce inflammation, thus reducing pain in the short-term [19,32,33]. In addition, magnesium chloride, which is unique to the PTMC roll-on, has been shown to be effectively absorbed through the dermis into muscle [34,35].

Topical analgesics are typically easy to self-apply. While this is a feature of many non-pharmacologic topicals, evidence of the effectiveness of the topical analgesics tested in this study is significantly lacking in the research literature. The results from the study demonstrate promising results for patients with myofascial pain. Since the topical analgesics are easy to use, they may improve the patients' self-care through increased compliance and help alleviate the painful symptoms they experience from MTrPs.

Many clinicians use these topical analgesics during multiple sessions or in combination with other proven therapies. Many clinicians theorize that the combination therapy or multiple session uses will result in long-term benefits. The intervention in this study was applied in one single session with only short-term outcomes measured. A similar study has been published on the outcomes from a single manual therapy session for patients with neck pain [36]. A clinical prediction rule has been formulated for single-session response to manual therapy for neck pain. Our findings show similar benefits compared with the manual therapy neck pain study and provides support for further research. Future studies with multiple sessions over several treatment days or in combination with other therapies are needed to validate the effects of the topical analgesics commonly used in clinical settings.

### Limitations

This study used participants with and without cervical spine and trapezius pain. This may have attenuated the outcomes measured in this study. Future research should separately evaluate pain and non-painful groups to eliminate any confounding factors.

Subjects were not excluded if they had prior experience, nor, was prior experience measured as an explanatory variable. However, our efforts to blind subjects to the nature of the topicals used should have reduced the effect of any prior treatment experience.

Only one trial of pressure algometery and cervical ranges of motion was obtained pre-post intervention for each patient. As there is likely to be some variability with examiner testing, multiple recordings should be obtained in future studies.

It is unknown if any of the significant results obtained immediately post-intervention would be sustained over a longer period of time. Future studies should measure longer term outcome measures, such as a few hours after treatment.

## Conclusions

This study demonstrated that some topical analgesic products do reduce myofascial pain or tenderness. The results of this study demonstrated that there were clinically important and significant differences between the topical analgesics tested. The BG, MM and the PTMC roll-on ointment demonstrated significant increases in pressure threshold levels. PTMC roll-on and BG were significantly superior to the placebo in the short-term reduction of myofascial tenderness. Furthermore, the PTMC roll-on demonstrated that it was significantly superior to the IH in the short-term reduction of myofascial tenderness.

There were no significant differences in range of motion testing for any of the topical analgesics. Future studies are needed to evaluate the effect of topical analgesics on the duration of effects of topical pain relief.

## Endnotes

^a^Professional Therapy Muscle Care^® ^Extra Strength Pain Relief Roll-on, Muscle Care Products (div. of Active & Innovative Inc.) Toronto, ON M4T 2V7

^b^MM Cream, Motion Medicine Inc., Calgary, AB T2Y 2Z7

^c^Bengay Ultra Strength Muscle Pain non-greasy, non-staining cream, Johnson & Johnson Inc. Markham, ON L3R 5L2, Canada.

^e^Icy Hot Extra Strength Pain Relieving Cream, Chattem Canada, Mississauga, ON L5N 2K7, Canada

^f^Biofreeze Pain Relieving Roll-on Gel with Ilex, Royal Cross, Concord, ON L4K 2L9, Canada

## Competing interests

The authors declare that they have no competing interests.

## Authors' contributions

DA have made substantial contributions to the acquisition of data, analysis and interpretation of data, have been involved in drafting the manuscript or revising it critically for important intellectual content and have given final approval of the version to be published. AH and CH have made substantial contributions to conception and design, acquisition of data, analysis and interpretation of data, have been involved in drafting the manuscript or revising it critically for important intellectual content and have given final approval of the version to be published. HV made substantial contributions to the design of this study as well as to the analyses and interpretations of the findings. He was substantially involved in drafting the manuscript or revising it critically for important intellectual content and has given final approval of the version to be published. All authors read and approved the final manuscript.
